# Supplementary material for: The Systems Biology Research Tool: evolvable open-source software
Source: BMC Syst Biol. 2008 Jun 29;2:55. doi: 10.1186/1752-0509-2-55 (PMC2446383; doi:10.1186/1752-0509-2-55)
Supplement: Additional file 1 — SBRT Archive. An archive of the current version of the Systems Biology Research Tool. [file 1752-0509-2-55-S1.zip › sbrt-1.4.0/doc/developers_guide/api/sbrt/kernel/class-use/MonitoredProcess.html]

Uses of Interface sbrt.kernel.MonitoredProcess


|  |  |  |
| --- | --- | --- |
| **Overview** \| **Package** \| **Class** \| **Use** \| **Tree** \| **Deprecated** \| **Index** \| **Help** | |  |
| PREV   NEXT | **FRAMES**    **NO FRAMES**     **All Classes** |  |


---


## **Uses of Interface sbrt.kernel.MonitoredProcess**

| Packages that use MonitoredProcess | |
| --- | --- |
| **sbrt.kernel.fba.proc** | Provides classes and interfaces for executing Flux Balance Analysis processes. |
| **sbrt.kernel.math.geom.proc** | Provides classes and interfaces for performing geometry-related processes. |
| **sbrt.kernel.math.graph\_theory.proc** | Provides classes and interfaces for performing graph theoretical processes. |
| **sbrt.kernel.math.stat.proc** | Provides classes and interfaces for performing statistical processes. |
| **sbrt.kernel.utilities.proc** | Provides classes and interfaces for performing utility processes. |
| **sbrt.shell.mng** | Provides classes and interfaces for creating process managers. |

Uses of MonitoredProcess in sbrt.kernel.fba.proc

| Classes in sbrt.kernel.fba.proc that implement MonitoredProcess | |
| --- | --- |
| `class` | `CatalystDeletionProcess`             This class is used to perform catalyst deletion experiments. |
| `class` | `CdHarFluxVectorProcess`             This class is used to perform coordinate direction hit-and-run flux vector experiments. |
| `class` | `CvCatalystDeletionProcess`             This class is used to perform constraint-variation catalyst deletion experiments. |
| `class` | `CvFbaOptimizationExperiment`             This class is used to perform constraint-variation FBA optimization experiments. |
| `class` | `CvFluxIntervalProcess`             This class is used to perform constraint variation flux interval experiments. |
| `class` | `CvObjectiveFunctionProcess`             This class is used to perform constraint-variation objective function experiments. |
| `class` | `CvRxnDeletionProcess`             This class is used to perform constraint-variation reaction deletion experiments. |
| `class` | `CycleIdProcess`             This class is used to execute steady state cycle identification processes. |
| `class` | `ExtremeCurrentProcess`             This class is used to perform extreme current experiments. |
| `class` | `FluxActivityProcess`             This class is used to perform flux activity experiments. |
| `class` | `FluxCapProcess`             This class is used to perform a flux cap identification. |
| `class` | `FluxIntervalProcess`             This class is used to perform flux interval experiments. |
| `class` | `FluxomeReducerProcess<T extends Fluxome>`             This class is used to execute fluxome reduction processes. |
| `class` | `FluxPlasticityProcess`             This class is used to perform flux plasticity experiments. |
| `class` | `ObjectiveFunctionAnalysis`             This class is used to perform objective function experiments. |
| `class` | `RandomConstraintsGeneratorV1`             This class is used to generate random flux constraints. |
| `class` | `RandomEnvGeneratorV1`             This class is used to generate random environments for use in flux balance analysis. |
| `class` | `RandomObjectiveGenerator`             This class is used to generate random objective functions. |
| `class` | `RxnDeletionProcess`             This class is used to perform reaction deletion experiments. |
| `class` | `StoichVariationProcess`             This class is used to perform stoichiometry-variation FBA optimizations. |
| `class` | `SvCdHarFluxVectorProcess`             This class is used to perform space variation coordinate direction hit-and-run flux vector experiments. |
| `class` | `SvInitialPointProcess`             This class is used to perform constraint variation initial point experiments. |

Uses of MonitoredProcess in sbrt.kernel.math.geom.proc

| Classes in sbrt.kernel.math.geom.proc that implement MonitoredProcess | |
| --- | --- |
| `class` | `CdHarProcess<V>`             This class is used to generate random, uniformly distributed points from the interior of a convex polytope. |

Uses of MonitoredProcess in sbrt.kernel.math.graph\_theory.proc

| Classes in sbrt.kernel.math.graph\_theory.proc that implement MonitoredProcess | |
| --- | --- |
| `class` | `PathIdProcess<N>`             This class is used to identify all the simple paths in a directed graph. |

Uses of MonitoredProcess in sbrt.kernel.math.stat.proc

| Classes in sbrt.kernel.math.stat.proc that implement MonitoredProcess | |
| --- | --- |
| `class` | `MultipleCorrelationAnalysis<V,T extends CorrResult>`             This class is used to perform multiple correlation analyses. |

Uses of MonitoredProcess in sbrt.kernel.utilities.proc

| Classes in sbrt.kernel.utilities.proc that implement MonitoredProcess | |
| --- | --- |
| `class` | `IntervalComparison<V>`             This class is used to compare sets of intervals. |
| `class` | `VectorComparison<V>`             This class is used to compare sets of intervals. |

Uses of MonitoredProcess in sbrt.shell.mng

Methods in sbrt.shell.mng with parameters of type MonitoredProcess

`static

<T> void`

`AbstractProcessManager.runProcess(MonitoredProcess<T> process,
ResultsManager<T> resultsManager,
java.io.PrintWriter out)`
  
          Runs the provided process using the provided results manager and prints
descriptive progress messages to the provided print writer.

---


|  |  |  |
| --- | --- | --- |
| **Overview** \| **Package** \| **Class** \| **Use** \| **Tree** \| **Deprecated** \| **Index** \| **Help** | |  |
| PREV   NEXT | **FRAMES**    **NO FRAMES**     **All Classes** |  |


---
